# Rapunzel Syndrome: A Rare Presentation with Giant Gastric Ulcer

**DOI:** 10.1155/2014/267319

**Published:** 2014-11-06

**Authors:** Antonios Athanasiou, Adamantios Michalinos, Demetrios Moris, Eleftherios Spartalis, Nikolaos Dimitrokallis, Vaios Kaminiotis, Demetrios Oikonomou, John Griniatsos, Evangelos Felekouras

**Affiliations:** First Department of Surgery, Laikon General Hospital, National and Kapodistrian University of Athens, Agiou Thoma Street 17, Attiki, 11527 Athens, Greece

## Abstract

The Rapunzel syndrome refers to an uncommon and rare form of trichobezoar that extends past the stomach into the small intestines. The Rapunzel syndrome is usually found in young female patients with a history of psychiatric disorders, mainly trichotillomania and trichophagia. We describe a case of Rapunzel syndrome in a 15-year-old girl who presented with abdominal pain, vomiting, and weight loss. We performed a surgical laparotomy and successfully removed a huge trichobezoar extending into the small intestine.

## 1. Introduction

A bezoar is a mass of foreign and intrinsic material found in the gastrointestinal tract, most commonly in the stomach. The term bezoar derives from the Arabic word “Badzehr” or the Persian word “padzhar” which means antidote [1]. Bezoars can be classified in six types: phytobezoars (composed of indigestible plant material), trichobezoars (hairball or hair-like fibers), lithobezoars (fragments of small stones, pebbles, or gravel stones), pharmacobezoar (mostly tablets or semiliquid masses of drugs), plasticobezoars (plastic), and lactobezoars (inspissated milk) [2]. The most frequent type of bezoar in adults is phytobezoar, while trichobezoar is more often found in children and teenage girls [3]. In contrast to most types of bezoars, trichobezoars are usually observed in individuals linked to pica and other psychiatric conditions, such as emotional disturbance, learning disabilities, and history of neglect or mental retardation. These individuals pull out their own hair and swallow it, processes referred to as trichotillomania and trichophagia [4, 5]. A rare manifestation of trichobezoar is Rapunzel syndrome (RS) which occurs when the bezoar extends into the small intestine. Common presenting symptoms of RS are abdominal pain, vomiting, nausea, weight loss, malnutrition, hematemesis, diarrhea, or constipation [4]. Here we present a rare case of RS in a 15-year-old girl, who presented with epigastric pain and malabsorption-related complications.

## 2. Case Presentation

A 15-year-old girl was admitted to our hospital with a 2-month history of appetite loss and moderate weight loss. Episodic abdominal pain and intermittent vomiting worsened after meals. According to her parents, the girl pulled out her own hair and swallowed it, since the age of 5. On examination, the patient was anemic with normal vital signs. Physical examination of her abdomen revealed a large and firm palpable mass from epigastrium to the periumbilical region and hyperactive bowel sounds.

**Figure 1:**
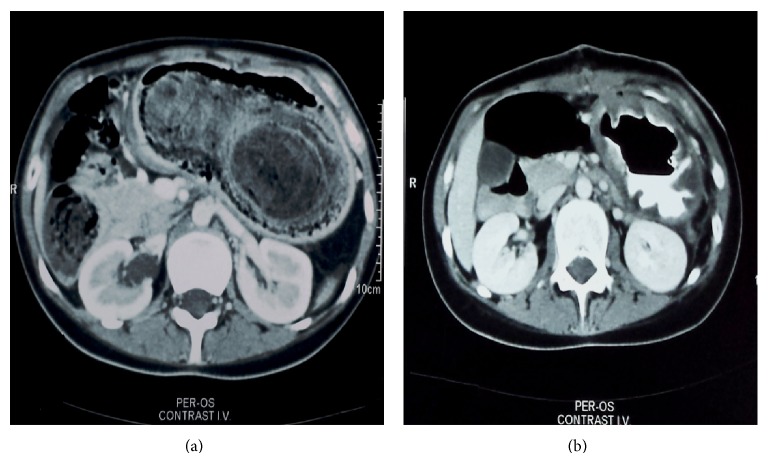
CT scan of the abdomen before the operation revealing a giant trichobezoar in the stomach and extending to the duodenum (a) and a CT scan of the abdomen 5 days after the operation showed massive dilation of the stomach (b).

**Figure 2:**
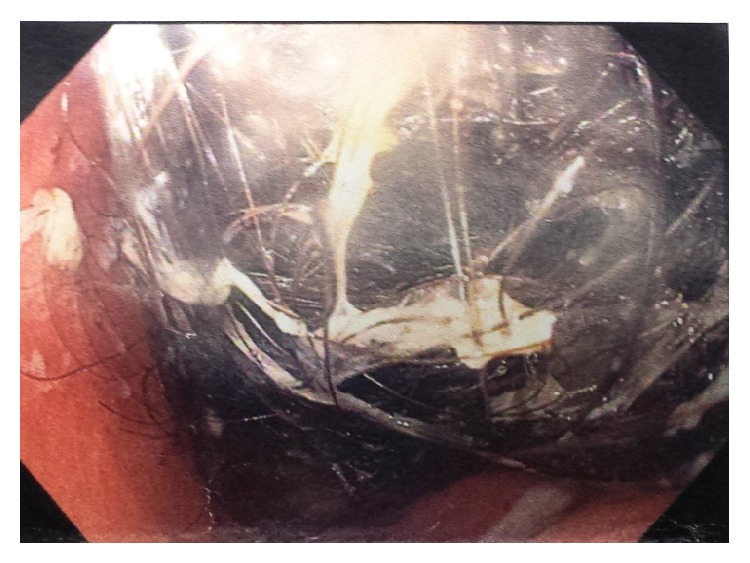
Endoscopic view of an intragastric trichobezoar.

Her complete blood picture revealed notable microcytic anemia, with a hemoglobin of 8.6 gm/dL, a mean corpuscular volume (MCV) of 56.4 *μ*m^3^, and a mean corpuscular hemoglobin (MCH) of 17.7 pgm, while the rest of the laboratory test findings were all within normal limits. On further evaluation, an ultrasound of the abdomen confirmed the presence of a large intragastric mass and an abdominal computed tomography (CT) scan revealed a huge hypodense and heterogeneous gastric mass, appearing as a mesh, with multiple air bubbles seen within and surrounding the mass (Figure 1(a)). The mass filled the gastric fundus and duodenum and extended into the first centimeters of the jejunum. Finally, an upper gastrointestinal endoscopy was performed and revealed the presence of a giant trichobezoar, extending from the distal esophagus and occluding the pylorus (Figure 2).

**Figure 3:**
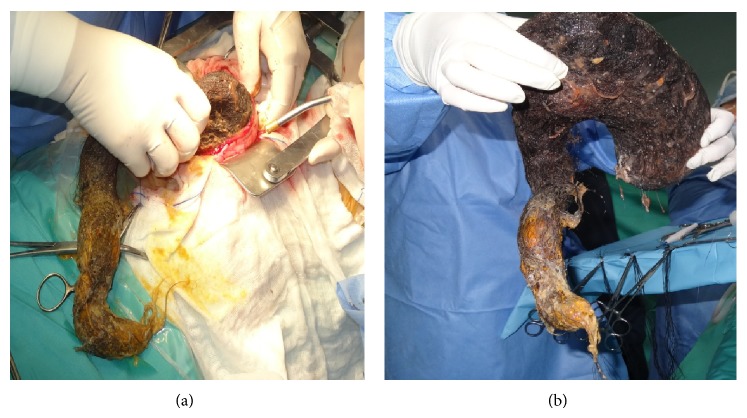
The trichobezoar has been extracted through the longitudinal gastrotomy (a). Specimen of the Rapunzel syndrome (b).

**Figure 4:**
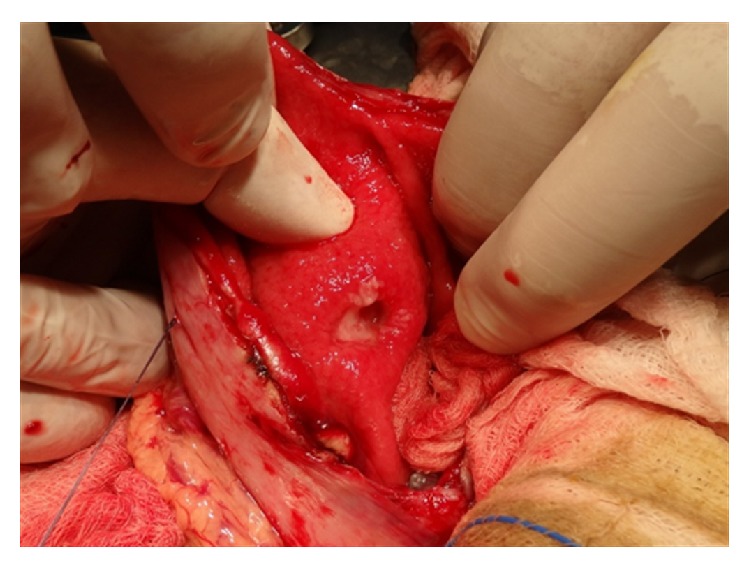
Prepyloric gastric ulcers.

Due to the bulky size of the trichobezoar, we decided that the most efficient and safe way of removing it was an elective laparotomy. Upper midline incision was performed and a longitudinal gastrotomy was made in the anterior wall of the distended stomach. The trichobezoar was identified and extracted (Figure 3(a)). The mass extended to proximal part of duodenum and the first centimeters of the jejunum. The mass weighed 1023 gr and measured 25 × 11 × 6 cm (Figure 3(b)). After the removal of the trichobezoar, the presence of 2 prepyloric gastric ulcers was revealed, from which biopsy specimens were taken (Figure 4). An intraoperative upper GI endoscopy was done in order to make sure that on the one hand, other abnormalities were not existed and on the other hand, the mass was fully removed. The ulcers were oversewed by using PDS 3.0 and the gastrotomy was closed in two layers by PDS 3 and 4.0. There were no postoperative complications and an abdominal CT scan 5 days after the operation proved to be normal (Figure 1(b)). The patient was discharged on the 6th postoperative day and the family was advised to visit a psychiatrist.

## 3. Discussion 

The RS, as a very rare and unusual form of trichobezoar, was originally described by Vaughan Jr. et al. in 1968 [6]. Despite the fact that incidence of trichobezoar is reported to be very low (0.4%) [7], the actual rate is unknown due to the fact that this syndrome is generally seen in people with psychiatric disorders. Furthermore, there is no accurate data on how many of the patients with trichophagia develop trichobezoars. While there are studies supporting that there is no development of trichobezoars in a number of patients with trichotillomania [5], a number of other studies report this rate as high as 25% [8].

The signs and symptoms which characterize the RS depend on the size of the trichobezoar and the presence of complications. RS is typically characterised by signs and symptoms of gastric outlet obstruction or malabsorption-related complications. According to Naik et al. [4], the most common presentations are abdominal pain (37%), nausea and vomiting (33.3%), obstruction (25.9%), and peritonitis (18.3%). Uncommonly though, patients have also presented with weight loss (7.4%), anorexia, hematemesis, and intussusceptions (7.4%). In our case, the patient was presented with trichophagia from the age of 5, while the main symptom, which has led the patient's parents to the hospital, was the abdominal pain and the intermittent vomiting that worsened after meals.

Upper gastrointestinal endoscopy is considered to be the gold standard for the diagnosis of the trichobezoar; however, it does not prove the existence of the RS. The abdominal CT scan is the most accurate imaging test concerning the presence of trichobezoars, since it demonstrates heterogeneous, mottled intraluminal mass with low attenuation and air trapping [9]. Furthermore, it can track with great detail the extension of the trichobezoar's tail to the gastrointestinal tract. Abdominal ultrasound and barium meal (with the characteristic honeycomb appearance) can prove to be useful. Management options for the treatment of the RS include surgical removal by laparotomy or laparoscopically [10]. Laparotomy is widely considered as the treatment of choice for complicated trichobezoar due to several significant reasons, including among others the high success rate, the relatively low complication rate, and the simple nature of the operation [10]. Moreover, it must be noted that the successful removal of large trichobezoars by laparotomy combined with anterior gastrotomy is confirmed by a retrospective analysis of 34 cases published in 2005 [11]. In the present case, due to the bulky size of the trichobezoar, we decided that the most efficient and safe choice for the patient was an elective laparotomy.

Recurrence of RS is extremely rare, with the total number of recurrences reported in the literature, being 3 [12]. The most common characteristic of the referred recurrences was that these patients defaulted follow-up after a few months and consequently did not complete the psychiatric treatments. In order to decrease recurrence, patients should receive psychiatric/psychological support after the surgical treatment. It should be kept in mind that the regular long-term psychiatric follow-up might prevent recurrences, although these are rare. In addition, despite the fact that studies of the pharmacotherapy of the trichotillomania remain inconsistent, recurrence seems to be avoided in some patients after pharmacotherapy [13].

In conclusion, despite the fact that the RS is an uncommon disorder, it should be included in the differential diagnosis of young female patients with a history of trichophagia and trichotillomania, chronic abdominal pain, nausea, and vomiting. As far as the diagnosis is concerned, it can be easily made with the use of endoscopy and abdominal CT scan, with the latter being the most accurate imaging test concerning the presence of trichobezoars. Management options for the treatment of the RS include surgical removal by laparotomy or laparoscopically, while laparotomy is widely considered as the treatment of choice for complicated trichobezoars. Recurrence of RS is extremely rare; however long-term psychiatric/psychological support and follow-up should be performed in order to prevent any recurrences.
